# Diagnostic Value of Dynamic Contrast-Enhanced Magnetic Resonance Imaging in the Evaluation of the Biliary Obstruction

**DOI:** 10.1100/2012/731089

**Published:** 2012-03-12

**Authors:** Mehmet Bilgin, Hüseyin Toprak, Mehmet Burgazli, S. Sennur Bilgin, Ritvan Chasan, Ali Erdogan, Cem Balcı

**Affiliations:** ^1^Department of Radiology, Medical Faculty, Bezmialem Vakif University, 34093 Istanbul, Turkey; ^2^Department of Internal Medicine, Justus-Liebig-University Hospital, 35392 Giessen, Germany; ^3^Department of Radiology, Justus-Liebig-University Hospital, 35392 Giessen, Germany; ^4^Department of Radiology, Istanbul Bilim University School of Medicine, 34340 Istanbul, Turkey

## Abstract

*Purpose*. In this study, our purpose was to investigate the diagnostic efficacy of the dynamic contrast-enhanced magnetic resonance imaging (MRI) method in the patients with bile duct obstruction. *Materials and Methods*. 108 consecutive patients (53 men, 55 women, mean age; 55.77 ± 14.62, range 18–86 years) were included in this study. All the patients underwent conventional upper abdomen MRI using intravenous contrast material (Gd-DTPA) and MRCP in 1.5 Tesla MRI scanner. MRCP images were evaluated together with the T1 and T2w images, and both biliary ducts and surrounding tissues were examined for possible pathologies that may cause obstruction. *Results*. MRI/MRCP findings compared with final diagnoses, MRI/MRCP in the demonstration of bile duct obstruction sensitivity 96%, the specificity 100%, and accuracy 96.3%, in the detection of presence and level of obstruction, the sensitivity 96.7%, specificity 100%, and accuracy 97.2%, in the diagnosis of choledocholithiasis, the sensitivity 82.3%, specificity 96%, and accuracy 91.7%, and in the determination of the character of the stenosis, sensitivity 95.6%, specificity 91.3%, and accuracy 94.5% were found. *Conclusion*. The combination of dynamic contrast-enhanced MRI and MRCP techniques in patients with suspected biliary obstruction gives the detailed information about the presence of obstruction, location, and causes and is a highly specific and sensitive method.

## 1. Introduction

Evaluation of biliary obstructions is still a common clinical problem. The first problem is often to distinguish intrahepatic and extrahepatic obstruction and to reveal the character of benign or malignant obstruction. Choledocholithiasis and pancreatobiliary malignancies are the most frequent cause of extrahepatic obstruction. In addition, benign strictures, chronic pancreatitis, papillary stenosis, metastatic lymph nodes in liver hilus, and primary sclerosing cholangitis may lead to bile duct obstruction. In most cases, with medical history, physical examination, and clinical and laboratory data, the presence of bile duct obstruction could be determined. However, imaging modalities are needed to fully evaluate the biliary obstruction. With these imaging modalities, the presence, location, and causes of obstruction are determined, and this forms the basis of appropriate treatment plan. Although abdominal USG and CT are the first performed imaging methods, for definite diagnosis, direct cholangiographic methods such as ERCP ve PTC are commonly referred [1–5].

 ERCP, since first appearance in 1970, in the evaluation of biliary tree, also protects its therapeutic feature and continues to be gold standard imaging method. But, today, in addition to high diagnostic accuracy of MRCP, as an invasive methods the morbidity and mortality rate of ERCP reaches about 7% and 1%, and this limits the use of ERCP for diagnosis. Also, in the case of hepaticojejunostomy and choledochojejunostomy, ERCP cannot be performed; in the case of gastric resection, retroperitoneal neoplasm, duodenal diverticulum, and ampullary edema, performing ERCP is hard. ERCP, dependent on the practitioner, in some of the cases is failed (3%–18%) or inadequate [6–11].

 In MRCP technique, reconstructed 3-dimensional coronal MIP images are similar with cholangiographic images obtained with percutaneous or endoscopic way. In addition, in situations where ERCP and PTC are failed or inadequate, MRCP is an alternative method in which diagnostic images can be obtained without the need for contrast material injection, ionizing radiation, and invasive procedure. In total occlusion, compared to ERCP and PTC, significant advantage of MRCP is, MRCP can demonstrate the upper and lower section of the biliary duct at obstruction and real size of the obstruction. In ERCP and PTC, the use of high pressure contrast material to overcome the obstruction may cause the perception of more dilatation. MRCP reflects the natural state of the channel system [6–13].

 Although in the evaluation of biliary obstruction, the addition of T1 w and T2 w images and contrast-enhanced images to MRCP increases the cost, the addition of them increases the diagnostic efficacy by providing more detailed information about extraductal pathologies and adjacent anatomical structures such as liver and pancreas as well as the character and spread of the tumor. There were many studies that revealed the diagnostic efficacy of MRCP in bile duct obstruction, but there were few studies in which both dynamic contrast-enhanced MRI and MRCP were used for this purpose [3, 14–16].

 In this study, diagnostic efficacy of the combined use of MRCP and contrast-enhanced MRI methods to evaluate the patients with bile duct obstruction was explored.

## 2. Materials and Methods

Data about patients with clinical symptoms and laboratory findings (increase in bilirubin and alkaline phosphatase) consistent with bile duct obstruction who were presented to our hospital clinics between March, 2005 and January, 2010 were retrospectively reviewed. 108 patients (55 women, 53 men, mean age 18–86 years, mean age 55.77 ± 14.62 years) in whom MRI/MRCP optimally performed and ERCP and/or PTC was performed in order to verify imaging findings within 1–7 days or with surgery definitive diagnosis had been achieved were included in this study. All patients' files were reviewed for clinical, laboratory, and interventional radiological examinations that had performed to patients; if present, surgical and histopathological results were reviewed also. As the reference imaging method ERCP was performed in 71 patients, PTC was performed in 21 patients and both of them were performed in 6 patients. In patients in whom interventional imaging methods were not performed, the exact diagnosis reached by surgical results. In all 23 patients with malignant obstruction, the definite diagnosis was obtained with biopsies taken during the surgical operation or interventional procedure. In all cases, MRI/MRCP findings were compared to final diagnoses obtained. This study was retrospective study and accordant with health insurance and responsibility act. Corporate Audit Committe approved the study, and informed consent was not required because of the retrospective design of the study.

### 2.1. MR Imaging

All patients underwent combined MRI of the upper abdomen and MRCP. MRI was performed in a 1.5 Tesla MR scanner (Intera, Philips Medical Systems, Best, The Netherlands) with the use of four-element quadrature phased-array surface coil. The standard upper abdomen MRI protocol consisted of following imaging sequences and parameters: T1-weighted spoiled gradient echo in dual phase (TR/TE/FA; 140–170 msec/4.4–2.2 msec/70°), T1-weighted fat-saturated 2D spoiled gradient echo (TR/TE/FA; 160–180 msec/4.3 msec/70°) and T2-weighted fat-saturated echo train fast spin echo (TR/TE; ∞/80 msec). All images were obtained in axial plane with 6 mm section thickness and 160–190 × 256 matrix with the SENSE factor of two. MRCP images were obtained using 2D breath-hold T2-weighted fat-saturated single shot fast spin echo (ssFSE) images (TR/TE, ∞/1200 msec) with a slab thickness of 30–50 mm in 12 paracoronal planes comprising an angle of 360°. For the acquisition of each separate plane, patients held their breath for five seconds. Gd-DTPA (Magnevist, Schering, Berlin, Germany) was injected in a dose of 0.1 mmol/kg of body weight as a bolus injection at 2 mL/sec using a power injector (Medrad Indianola, PA, USA). Images were acquired at 18 sec (arterial dominant phase), 45 sec (portal venous phase), and 90 sec (late venous phase) after contrast administration. For serial contrast-enhanced images T1-weighted fat-saturated 2D spoiled gradient echo sequence was used.

### 2.2. Image Analysis

All the images of the patients were loaded to a work station (Workstation; Easyvision, Philips Medical Systems, Best, The Netherlands). Thick and thin MRCP and basal MRI images were reviewed by 2 radiologists who were unaware of the final diagnosis in consensus. In our study, the patients' images, whether there was dilatation and obstruction of biliary ducts, if any, the nature (benign or malignant) and location of obstruction, the presence of stone in the biliary ducts, the gallbladder status, tumor and similar pathologies that could lead to obstruction in surrounding tissues were evaluated. Extraductal structures were evaluated with axial and if necessary coronal plan T1 and T2w slices. For extrahepatic bile ducts, 7 mm diameter common biliary duct or more in the widest diameter (in patients with cholecystectomy 10 mm) and for intrahepatic bile ducts, 3 mm diameter or more was accepted as dilatation [17, 18]. The presence of stones in the bile ducts and gallbladder was investigated. The stones were evaluated as round, oval, and amorphous signal-void areas. In the evaluation, the number and size of the stones were ignored, just the presence or absence of stones indicated. The presence of stenosis in bile ducts, location of stenosis, and the nature of stenosis were examined. Level of stenosis was grouped into 3 levels as intrahepatic and/or hilar, extrahepatic-suprapancreatic, and intrapancreatic and/or periampullary. In strictures abrupt termination, long segment involvement, and irregular margin were accepted as malignancy criteria; smooth gradual tapering, short segment involvement and regular margin were accepted as benignity criteria. In addition, the demonstration of hyperintense areas around the stenosis on T2w images and the contrast enhancement of these areas on T1w images was accepted as a criterion in favor of tumor [3, 14, 18–24]. For malignant obstructions, in addition to axial images, when it was necessary coronal plan images were also obtained. If a tumor was present, the tumor mass, size, the relationship with surrounding tissues, and contrast-enhancement pattern were evaluated.

## 3. Results

According to final diagnoses, in 34 of 108 patients (31.5%) bile duct obstruction was due to choledocholithiasis, in 23 of them (21.3%), bile duct obstruction was due to malignant obstruction, in 34 of them (31.5%), bile duct obstruction was due to various benign strictures; totally, in 91 patients (84%), it was found. In 17 patients (16%), obstruction was not seen. According to final diagnoses, in 97 of 108 patients (90%), there was bile duct dilatation. Dilatation of bile ducts was observed in 6 patients although any pathology had not been found to explain this. In 11 patients (10%), no dilatation was seen.

**Figure 1:**
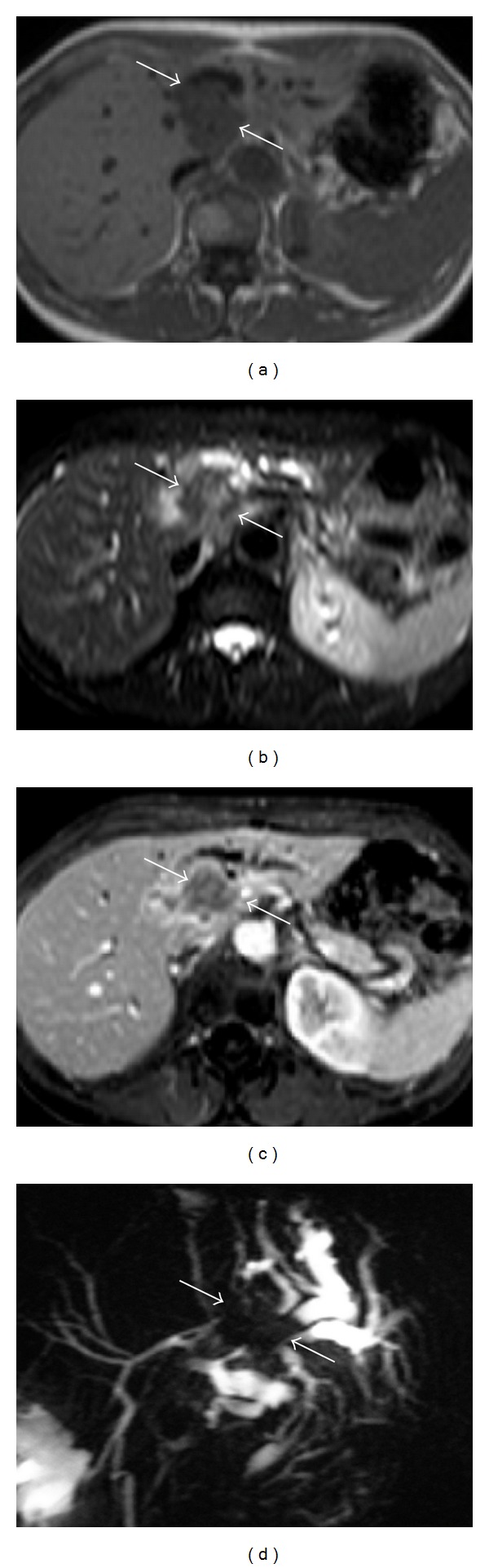
This figure demonstrates a mass lesion located in the left lobe of the liver close to hilus (a) hypointense on T1-weighted MR image, (b) heterogenous hyperintense on T2-weighted image, and (c) heterogenous contrast enhancement on contrast-enhanced fat-saturated T1-weighted image. (d) MRCP image demonstrates the mass that originated from left main bile duct and causes significant bile duct dilatation at the periphery of the liver (arrows). The lesion was considered to be a cholangiocarcinoma with MRI and MRCP findings and also confirmed surgically.

 In 3 of 34 patients with choledocholithiasis, stones in intrahepatic bile ducts were also present at the same time. 36 of 108 patients had cholecystectomy operation, in 7 of them, stones were detected in common bile duct. In 37 of 72 patients, in whom the gallbladder were present, stone/stones were present in gallbladder, and in 27 of these 37 patients, choledocholithiasis was also observed at the same time. In 34 patients, the cause of the obstruction was determined as benign stricture. In 10 of these patients, iatrogenic bile duct stricture that developed due to cholecystectomy operation, in 8 of them papillary stenosis (fibrotic stenosis and Oddi sphincter dysfunction), in 7 strictures due to inflammatory conditions (e.g., sclerosing cholangitis), in 7 chronic pancreatitis, in 2 Mirizzi syndrome, in 2 duodenal diverticula, and in 1 choleduct cyst were found (In 3 patients, more than one benign stricture were present). In 23 patients with malignant obstruction, cholangiocarcinoma in 6 patients (Figure 1), hepatocellular carcinoma in 2 patients, liver metastasis in 4 patients, metastasis to pancreas head in 2 patients, pancreas head adenocarcinoma in 4 patients, ampullary/periampullary carcinoma in 4 patients, and gallbladder carcinoma in 1 patient were detected.

On the basis of reference results, in 93 of 97 patients with bile duct dilatation, dilatation could be accurately detected using MRI/MRCP; 4 patients had false negative diagnosis. In 88 of 91 patients in whom the obstruction were detected, the presence and location of obstruction were detected accurately; on the other hand, in 3 patients, the presence of obstruction and therefore the level of obstruction could not be detected (Table 1). Although in 34 patients with choledocholithiasis calculi were detected accurately using MRCP in 28 patients, in 6 patients, false negative and in 3 patients false positive diagnosis were obtained. According to final diagnoses, in 23 patients malignant strictures were found as the cause of obstruction, 21 of these patients had accurate diagnosis, but 2 had false negative diagnosis; in 68 patients with benign obstruction, the accurate diagnosis was obtained in 65 of them, but 3 patients were misdiagnosed as malignant obstruction.

 When MRI/MRCP findings compared to final diagnoses, the sensitivity of MRI/MRCP in demonstrating bile duct dilatation was 96%, specificity was 100%, and accuracy was 96.3%, in the detection of the presence of obstruction, the sensitivity was 96.7%, specificity was 100%, and accuracy was 97.2%, in the diagnosis of choledocholithiasis, sensitivity was 82.3%, specificity was 96%, and accuracy was 91.7%, in the characterization of stenosis, sensitivity was 95.6%, specificity was 91.3%, and accuracy was 94.5%. Imaging findings that were obtained using MRI and MRCP, in comparison with final diagnoses, are presented in Table 2.

## 4. Discussion

In our study, intravenous contrast-enhanced MRI and MRCP images were evaluated together; in the determination of dilatation of bile ducts, choledocholithiasis, the presence of obstruction, location, and the nature of obstruction, high sensitivity and specificity were obtained.

 MRCP can show the presence of bile duct obstruction with 91%–100% accuracy [12, 22, 23]. In a study, in determining biliary obstruction with the combined use of MRI and MRCP, the sensitivity was found 82.3%, the specificity was found 93.8%, and accuracy was found 89% [14]. In our study which was similar to this study, in the determination of the presence of the obstruction, sensitivity was found 96.7%, specificity was found, 100% and accuracy was found 97.2%. In 3 patients, false negative results were obtained. In all of them, millimetric calculi in distal common bile duct and the obstruction were caused by them could not be revealed with MRI/MRCP, but with ERCP, these calculi could be detected.

 MRCP can show the level of obstruction with a rate of 85%–100% [12, 14, 21–23]. In our study, the accuracy in determining the level of stenosis was found 97.2%. False results came from 3 patients with choledocholithiasis mentioned above. In these patients, with MRCP, the small calculi could not be shown thus the stenosis level could not be determined; therefore, false negative results were obtained. Our results in detection of bile duct obstruction and in the determination of the level of obstruction were quite compatible with the results of the literature.

 MRCP can show the intra- and extrahepatic bile duct dilatation with high accuracy. Bile duct dilatation could have been determined with 94%–100% accuracy in recent studies [16, 18]. In our study, in the detection of the presence of dilatation, the sensitivity of MRCP was found 96%, specificity was found 100%, and accuracy was found 96.3%, and this was compatible with the results of literature. In our study, in the detection of dilatation, in 4 patients false negative results were obtained when MR images were compared to images based on the ERCP images. False negative results in these patients, as mentioned in many studies, was caused by the application of contrast material with high pressure to overcome the obstruction, and bile ducts were perceived wider than normal, and so, the false positiveness of ERCP played a role in this case. In these 4 patients, in the followup with USG, there was no dilatation in intra- and extrahepatic bile ducts.

**Figure 2:**
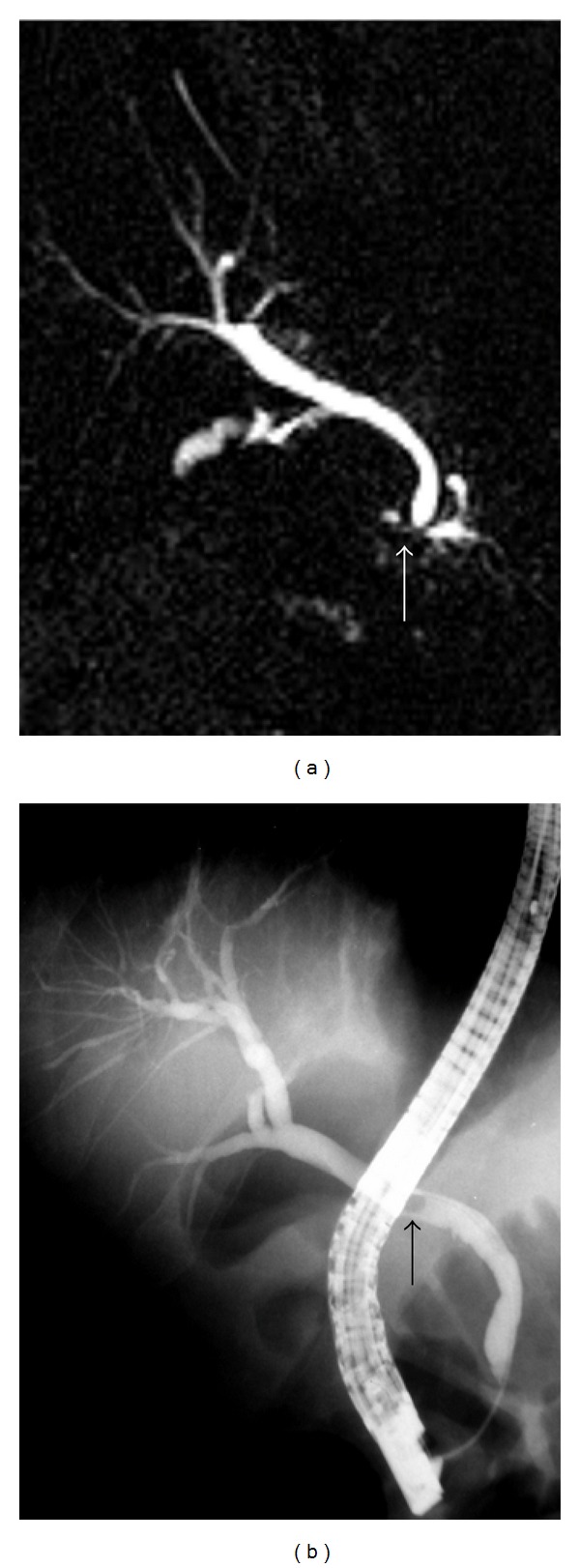
MRCP image demonstrates (a) a calculus (white arrow) located in the distal common bile that does not cause bile duct dilatation. This calculus was not detected during the MRI examination and received a false negative diagnosis. (b) ERCP image demonstrates the calculus (black arrow). This calculus was extracted during the procedure.

**Figure 3:**
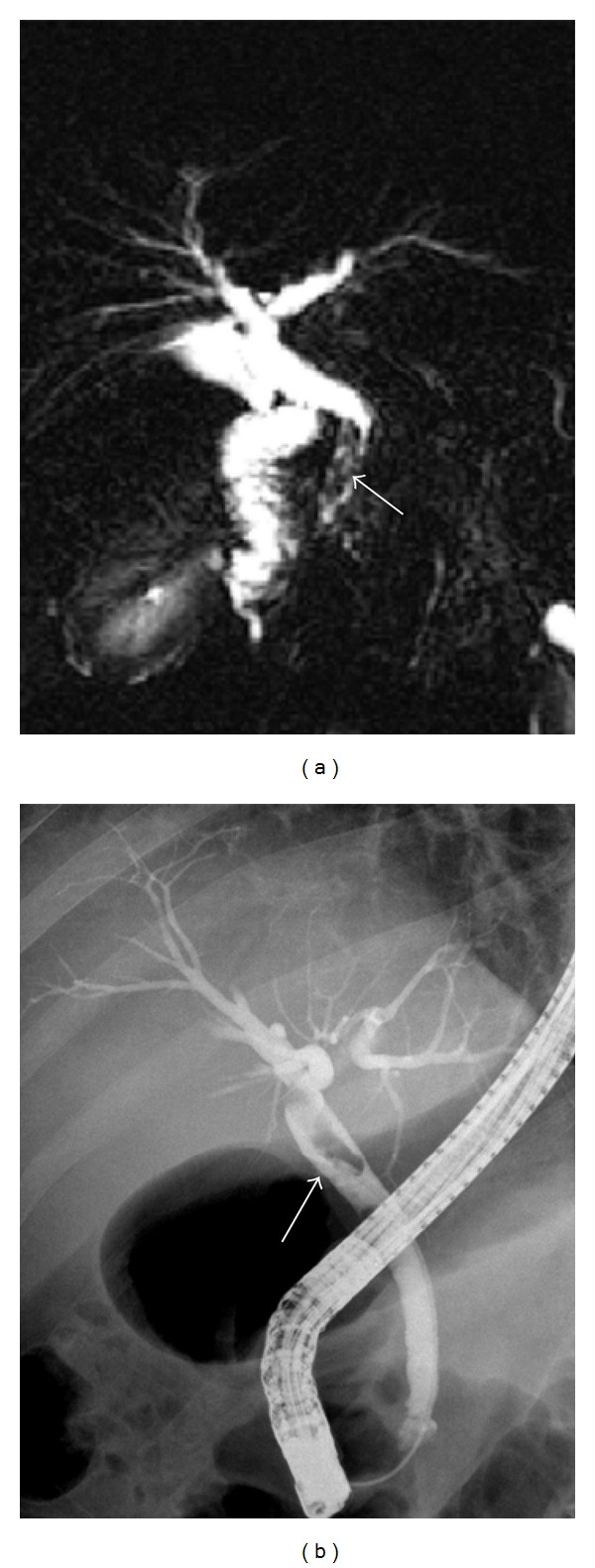
MRCP image demonstrates an irregular heterogenous hypointense region in the distal common bile duct (arrow). On MRI, this region evaluated as benign pathology such as calculus and sludge. ERCP image demonstrates a filling defect in this region (arrow). This filling defect was determined as Fasciola hepatica and extracted during the procedure.

 Gallstones in common bile duct are the most common cause of the bile duct obstruction [16]. Gallstones on MRI, regardless of their chemical composition, commonly seen as signal-void areas in lumen, but sometimes they may be overlooked. In the determination of gallstone, the sensitivity of MRCP depends on the size of the stone [5, 16]. In the literature, 88%–100% sensitivity and 89%–100% specificity rates have been reported in diagnosis of choledocholithiasis with MRCP [12, 20–25]. In our study, the sensitivity of MRI/MRCP in the diagnosis of choledocholithiasis was found 82.3%, the specificity was found 92.2%, and accuracy was 91.7%; this was compatible with the results of the literature. Sensitivity was relatively low in our study, because in 6 of 34 patients with choledocholithiasis, the calculi could not be detected with MRCP. In 5 of these patients, the size of calculi detected and removed with ERCP was 1–5 mm. When MRCP images were retrospectively reexamined, it was understood that in 3 patients, any stone was seen, in 1 patient, 8 mm calculus in distal choleduct was interpreted as benign stricture, in 1 patient a few 1–3 millimetric calculi at papillary level and in 1 patient 5 millimeter calculus at papilla were overlooked (Figure 2). In the literature, authors have been reported that when there is inadequate bile which forms a contrast around the stone, stone may not be seen or small stones may be hidden because of partial volume effect in hyperintense bile [20, 21]. The most difficult cases in diagnosis with MRCP were the calculi impacted at papilla or millimetric calculi. To see the millimetric calculi, in addition to MIP reconstructed images, base images also must be investigated in detail. However, if the calculi cannot be demonstrated with MRCP and the suspicion of calculi continues, invasive imaging techniques may be needed for definitive diagnosis [24, 25]. In our study, false negative results were obtained in 3 patients. In one of these patients, benign strictures at the middle level of common bile duct, and in the other patient, benign stricture at distal common bile duct were evaluated as calculus. In the 3rd patient, the Fasciola hepatica parasite settled in the distal common bile duct which was extracted by papillotomy during ERCP was interpreted in favor of calculus (Figure 3). In MRCP, strictures, intraductal air bubbles, protein plugs, blood clots, parasites, foreign bodies, surgical clips, and pulsation artifacts caused by arteries near the neighborhood were seen as signal void areas and may receive the wrong diagnosis as a calculus [20–24].

Stenoses occurred after cholecystectomies (mostly laparoscopic) constitute a significant part of benign bile duct stenoses. In addition, bile duct stenoses may be developed as a complication of surgical operations such as gastric and liver resection, liver transplantation, portocaval shunt operation and interventional procedures such as ERCP. Primary sclerosing cholangitis, chronic pancreatitis, and papilla Vater stenosis may also cause bile duct stenosis. Factors such as papillary malformation, inflammation, and spasm secondary to irritation of stone may cause stenosis of papilla and may prevent the bile flow. Malignant strictures causing bile duct obstruction are caused by external compression and/or invasion secondary to pancreaticobiliary malignancies or metastases of other malignancies [1–5, 21]. In MRI/MRCP, benign strictures manifest themselves as smooth gradual tapering of stenosis, regular border, and short segment involvement; in spite of this, pancreaticobiliary malignant lesions manifest themselves as abrupt termination of stenosis, irregular border, and long segment involvement. In addition, hyperintense regions on T2w images around the stenosis or contrast enhancement on T1w images are other important criteria which show the tumoral lesions location and spread. Despite these criteria, MRCP may be insufficient in distinguishing the benign or malignant character of the stenosis from time to time [2, 3, 14, 18–26].

 In the literature, in differentiation between malignant and benign obstruction, accuracy rates of MRCP ranging between 30%–100% have been reported. Variability of rates may be caused by various sequences used in source images and evaluation of MRCP images without MRI slices. Some studies have shown that addition of T1w and T2w images into the examination increase the sensitivity in the evaluation of malignant lesions [12, 15, 18, 25, 26]. In one study, when only MRCP was used in the differentiation of benign and malignant biliary obstruction, sensitivity 64.7%, specificity 81.2%, and accuracy 74.4% were found; however, when both MRI and MRCP were used, sensitivity 82.3%, specificity 93.8%, and accuracy 89% were found [14]. In our study, in the differentiation of benign and malignant biliary obstruction, the sensitivity of MRCP/MRI method was found 95.6%, specificity was found 91.3%, and accuracy was found 94.5% and was compatible with the results of the literature. The reason why our results were higher than the literature might be caused by the positive contribution of intravenous contrast-enhanced MRI to the diagnosis which all the patients underwent.

**Figure 4:**
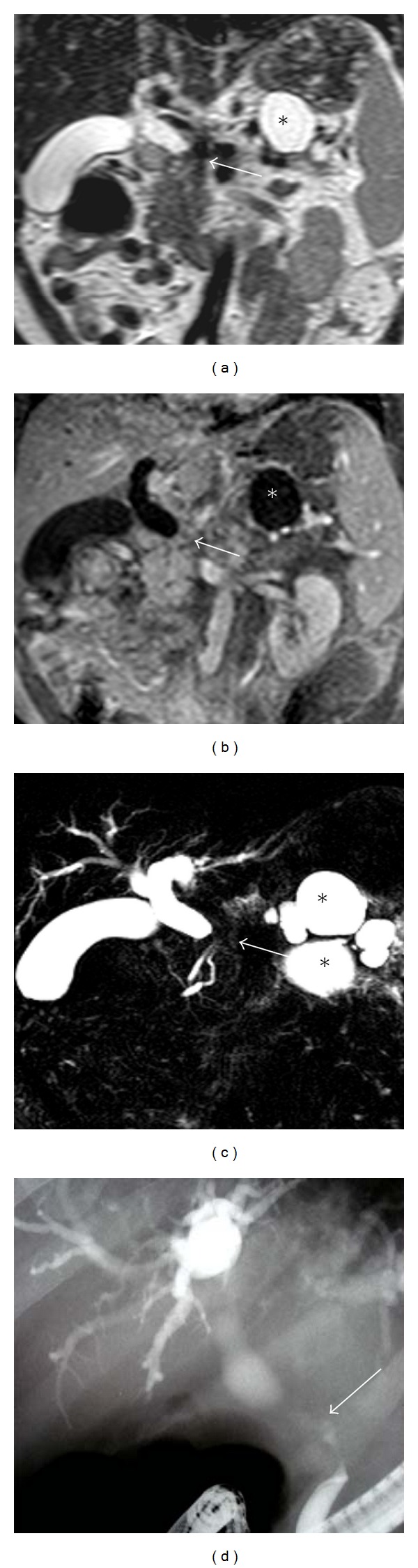
T2-weighted (a), contrast-enhanced T1-weighted MRI (b), and MRCP slices demonstrate significant stenosis in the common bile duct at the level of pancreas head (white arrow) and bile duct dilatation proximal to the stenosis. Any mass lesion is not seen at the stenosis level. On MRI and MRCP, the cause of stenosis considered as chronic pancreatitis because of pseudocysts located in corpus and tail of the pancreas (asterisk). (d) ERCP demonstrates the malignant character of stricture (black arrow), and the result of biopsy was adenocarcinoma of the pancreas.

**Figure 5:**
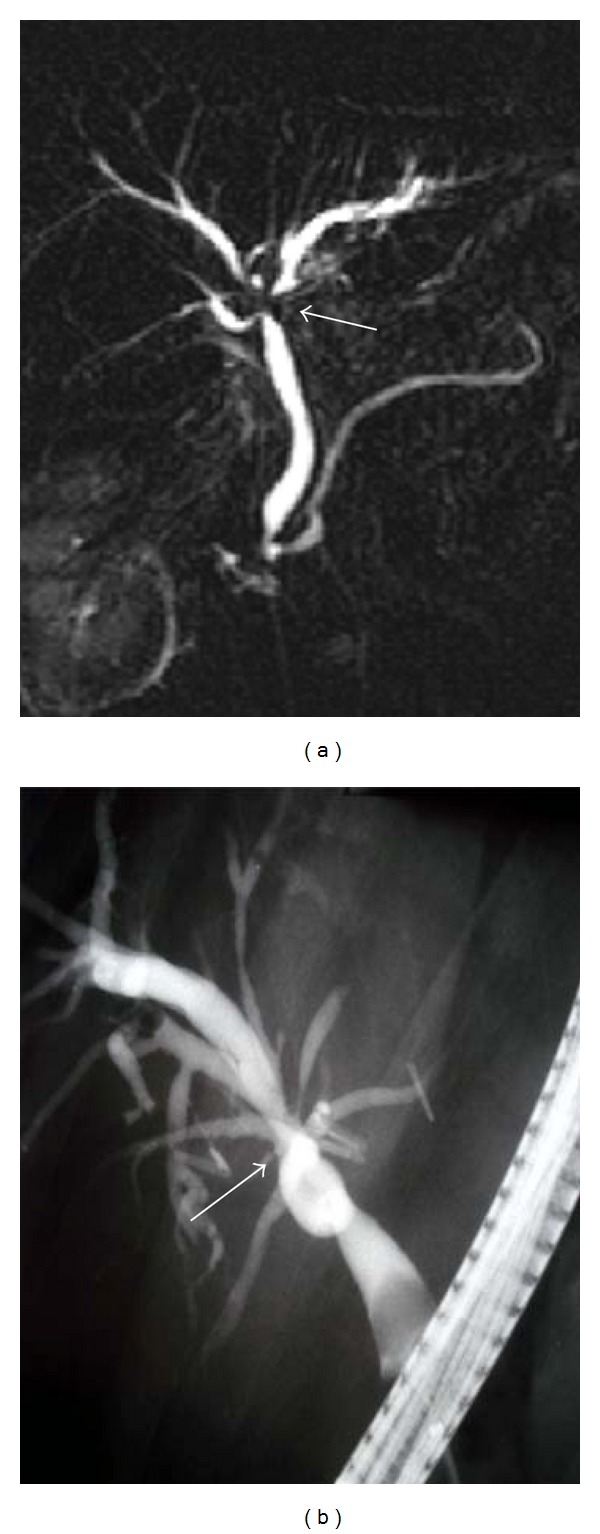
In a case who had undergone laparoscopic cholecystectomy operation 3 years ago, MRCP image demonstrates (a) stricture at hilar level (arrows) and mild dilatation of left main hepatic duct. Although this case had received false positive diagnosis as malignant stricture with MRI and MRCP findings, during ERCP, benign stricture developed secondary to cholecystectomy was diagnosed (arrows). Balloon dilatation and stent replacement was performed during ERCP procedure, and the result of biopsy was benign.

 In our study, 3 patients with malignant stricture were misdiagnosed as benign. In one of these patients, there was a pancreas head carcinoma in which a mass could not be demarcated on MR scans, no dilatation was seen in pancreatic ductus, and stenosis caused by pancreas head carcinoma was evaluated as benign stricture (Figure 4). In the literature, it has been reported that if there was no ductal dilatation, with MRI and MRCP, the diagnosis of pancreas carcinoma would be difficult [27, 28]. In another patient, stenosis at the papilla level was evaluated as inflammatory stenosis, but it was diagnosed as Ampulla Vateri tumor histopathologically. Obstruction at the level of ampulla can be caused by inflammatory stenosis, Oddi sphincter dysfunction, edema, stone, ampullary carcinoma, or pancreatitis. In the evaluation of lesions in this region, MRI/MRCP has a low performance due to artifact caused by intraaluminal gas located close to duodenal wall [13]. Therefore, ampullary region is a region that diagnostic mistakes encountered frequently, and if clinical suspicion continues, ERCP method in which ampulla visualized directly should be performed [28, 29]. In the third patient who had a wrong diagnosis, bilioenteric anastomosis had been performed because of chronic pancreatitis 10 years ago, and stenosis developed at anastomosis site was diagnosed as fibrotic stenosis. In this patient, in biopsy specimens obtained during the bile duct revision operation performed for renewing anastomosis, carcinoma was demonstrated. In a wide research serial of 1003 patients with bilioenteric anastomosis, it has been reported that cholangiocarcinoma may develop in the late period; therefore, possibility of malignancy should be considered in these kinds of anastomosis stenoses [30]. In our study, two patients with benign stricture had wrong diagnosis. In a patient with Mirizzi syndrome who had wrong positive diagnosis, irregular stenosis in proximal common bile duct was defined as malignant stricture on MRI/MRCP. In the other patient, benign stricture at hilar region developed secondary to cholecystectomy operation was interpreted as malignant (Figure 5). In some studies, authors have reported that in the differential diagnosis of benign or malignant stenoses at hilar region or proximal bile ducts experiencing difficulties [1, 19, 31]. On the other hand, in a recent study, the combination of MRI/MRCP in the evaluation of cholangiocarcinomas at hilar region has been reported to be the most reliable imaging method [32].

## 5. Conclusion

In our study in which both contrast-enhanced MRI and MRCP were used together, high sensitivity and specificity values were obtained in the evaluation of biliary obstruction. In the detection of small stones, in the evaluation of pathologies in ampullary and periampullary region, and in the differential diagnosis of benign and malignant lesions at hilar region with MRI/MRCP, sometimes difficulties may be seen. For definitive diagnosis in this case, invasive imaging procedures in which direct visualization of ampulla and biopsies can be performed during the procedure may be needed. In general, MRI/MRCP is a noninvasive imaging method with high accuracy rates in the evaluation of obstructive biliary tract pathologies may replace the diagnostic ERCP and PTC. 

##  Conflict of Interests

The authors do not have a direct financial relation with the commercial identity (e.g., “Magnevist, Schering”). They have no such conflict of interests.

## Figures and Tables

**Table 1:** The distribution of 98 different levels of the biliary tract obstruction (*n*: 91).

Level of obstruction	MRI/MRCP	Final diagnosis	MRI/MRCP accuracy (%)
Intrahepatic/hilar	30	30	97.2
Extrahepatic/suprapancreatic	19	19	
Intrapancreatic/periampullary	46	49	

**Table 2:** Signs evaluated with MRI/MRCP and comparison with final diagnoses.

Signs evaluated in bile duct	Result	MRI/MRCP	Final diagnosis	False negative	False positive	Sensit. (%)	Specif. (%)	Accuracy (%)
Dilatation (*n*: 108)	present	93	97	4	0	96	100	96.3
	absent	15	11					

Obstruction (*n*: 108)	present	88	91	3	0	96.7	100	97.2
	absent	20	17					

Cholelithiasis (*n*: 108)	present	31	34	6	3	82.3	96	91.7
	absent	77	74					

Nature of obstruction (*n*: 91)	Benign	67	68	3	2	95.6	91.3	94.5
	Malignant	24	23					

## References

[1] Schulz HJ (2008). Indeterminate bile duct strictures. *Gastroenterologe*.

[2] Soto JA, Yucel EK, Barish MA, Chuttani R, Ferrucci JT (1996). MR cholangiopancreatography after unsuccessful or incomplete ERCP. *Radiology*.

[3] Vogl TJ, Zeuzem S, Zangos S, Hammerstingl R (2009). Magnetresonanz-Cholangiopankreatikographie (MRCP) aus radiologischem und gastroenterologischem Blickwinkel. Magnetic resonance cholangiopancreatography (MRCP) from a radiological and gastroenterological perspective. *Deutsche Medizinische Wochenschrift*.

[4] Lubienski A, Duex M, Lubienski K, Blietz J, Kauffmann GW, Helmberger T (2005). Interventions for benign biliary strictures. *Radiologe*.

[5] Tse F, Barkun JS, Romagnuolo J, Friedman G, Bornstein JD, Barkun AN (2006). Nonoperative imaging techniques in suspected biliary tract obstruction. *HPB (Oxford)*.

[6] Filippone A, Ambrosini R, Fuschi M, Marinelli T, Pinto D, Maggialetti A (2003). Clinical impact of MR cholangiopancreatography in patients with biliary disease. *Radiologia Medica*.

[7] Glasbrenner B, Ardan M, Boeck W, Preclik G, Möller P, Adler G (1999). Prospective evaluation of brush cytology of biliary strictures during endoscopic retrograde cholangiopancreatography. *Endoscopy*.

[8] Buset M, Dunham F, Gulbis A, Boyazis M, Jeanmart J, Toussaint J (1981). Late complications of the diagnostic and operative endoscopy of the bile ducts and the pancreas. International study (author’s transl). *Acta Gastro-Enterologica Belgica*.

[9] Stiris MG, Tennoe B, Aadland E, Lunde OC (2000). MR Cholangiopancreatography and endoscopic retrograde cholangiopancreatography in patients with suspected common bile duct stones. *Acta Radiologica*.

[10] Rosch T, Meining A, Fruhmorgen S (2002 ). A prospective comparison of the diagnostic accuracy of ERCP, MRCP, CT, and EUS in biliary strictures. *Gastrointestinal Endoscopy*.

[11] Schumacher B (2006). Diagnostik und Therapie von Gallenwegserkrankungen—ERCP und MRCP. *Gastroenterologie Up2date*.

[12] Fulcher AS, Turner MA (2002). MR cholangiopancreatography. *Radiologic Clinics of North America*.

[13] David V, Reinhold C, Hochman M (1998). Pitfalls in the interpretation of MR cholangiopancreatography. *American Journal of Roentgenology*.

[14] Zhong L, Yao QY, Li L, Xu JR (2003). Imaging diagnosis of pancreato-biliary diseases: a control study. *World Journal of Gastroenterology*.

[15] Kim MJ, Mitchell DC, Ito K, Outwater EK (2000). Biliary dilatation: differentiation of benign from malignant causes—value of adding conventional MR imaging to MR cholangiopancreatography. *Radiology*.

[16] Zajaczek JEW, Keberle M (2005). Value of radiological methods in the diagnosis of biliary diseases. *Radiologe*.

[17] Park MS, Kim TK, Kim KW (2004). Differentiation of extrahepatic bile duct cholangiocarcinoma from benign stricture: findings at MRCP versus ERCP. *Radiology*.

[18] Baykara M, Erdoğan N, Özcan N (2006). Tıkanma tipi sarılığı olan olgularda manyetik rezonans kolanjiyopankreatografi bulgularının perkütan transhepatik kolanjiyografi bulgularıyla karşılaştırılması. *Erciyes Tıp Dergisi*.

[19] Dumonceau JM, Macias Gomez C, Casco C (2008). Grasp or brush for biliary sampling at endoscopic retrograde cholangiography? A blinded randomized controlled trial. *American Journal of Gastroenterology*.

[20] Bilgin M, Shaikh F, Semelka RC, Bilgin SS, Balci NC, Erdogan A (2009). Magnetic resonance imaging of gallbladder and biliary system. *Topics in Magnetic Resonance Imaging*.

[21] Kantarcı F, Selçuk D, Albayram S, Öğüt G (2003). Pankreatobilyer sistem patolojilerini değerlendirmede manyetik rezonans kolanjiyopankreatografi (MRCP)’nin tanı değeri. *Bilgisayarlı Tomografi Bülteni*.

[22] Halefoglu AM (2007). Magnetic resonance cholangiopancreatography: a useful tool in the evaluation of pancreatic and biliary disorders. *World Journal of Gastroenterology*.

[23] Pavone P, Laghi A, Panebianco V, Catalano C, Lobina L, Passariello R (1998). MR cholangiography: techniques and clinical applications. *European Radiology*.

[24] Brambs HJ, Hoffmann M, Pauls S (2005). Diagnosis and interventional therapy for ductal gallstones. *Radiologe*.

[25] Becker CD, Grossholz M, Becker M, Mentha G, De Peyer R, Terrier F (1997). Choledocholithiasis and bile duct stenosis: diagnostic accuracy of MR cholangiopancreatography. *Radiology*.

[26] Romagnuolo J, Bardou M, Rahme E, Joseph L, Reinhold C, Barkun AN (2003). Magnetic resonance cholangiopancreatography: a meta-analysis of test performance in suspected biliary disease. *Annals of Internal Medicine*.

[27] Hänninen EL, Amthauer H, Hosten N (2002). Prospective evaluation of pancreatic tumors: accuracy of MR imaging with MR cholangiopancreatography and MR angiography. *Radiology*.

[28] Diehl SJ, Lehmann KJ, Gaa J, Meier-Willersen HJ, Wendl K, Georgi M (1999). The value of magnetic resonance tomography (MRT), magnetic resonance cholangiopancreatography (MRCP) and endoscopic retrograde cholangiopancreatography (ERCP) in the diagnosis of pancreatic tumors. *Rofo*.

[29] Semelka RC, Kelekis NL, John G, Ascher SM, Burdeny D, Siegelman ES (1997). Ampullary carcinoma: demonstration by current MR techniques. *Journal of Magnetic Resonance Imaging*.

[30] Tocchi A, Mazzoni G, Liotta G, Lepre L, Cassini D, Miccini M (2001). Late development of bile duct cancer in patients who had biliary-enteric drainage for benign disease: a follow-up study of more than 1,000 patients. *Annals of Surgery*.

[31] Saluja SS, Sharma R, Pal S, Sahni P, Chattopadhyay TK (2007). Differentiation between benign and malignant hilar obstructions using laboratory and radiological investigations: a prospective study. *HPB (Oxford) Surgery*.

[32] Masselli G, Manfredi R, Vecchioli A, Gualdi G (2008). MR imaging and MR cholangiopancreatography in the preoperative evaluation of hilar cholangiocarcinoma: correlation with surgical and pathologic findings. *European Radiology*.

